# Predicting the daily number of patients for allergic diseases using *PM*_10_ concentration based on spatiotemporal graph convolutional networks

**DOI:** 10.1371/journal.pone.0304106

**Published:** 2024-06-13

**Authors:** Hyeon-Ju Jeon, Hyeon-Jin Jeon, Seung Ho Jeon

**Affiliations:** 1 Data Assimilation Group, Korea Institute of Atmospheric Prediction Systems (KIAPS), Seoul, Republic of Korea; 2 Department of Artificial Intelligence, Dongguk University, Seoul, Republic of Korea; 3 Department of Occupational and Environmental Medicine, Korea Industrial Health Association (KIHA), Seoul, Gyeonggi-do, Republic of Korea; The Catholic University of Korea, KOREA, REPUBLIC OF

## Abstract

Air pollution causes and exacerbates allergic diseases including asthma, allergic rhinitis, and atopic dermatitis. Precise prediction of the number of patients afflicted with these diseases and analysis of the environmental conditions that contribute to disease outbreaks play crucial roles in the effective management of hospital services. Therefore, this study aims to predict the daily number of patients with these allergic diseases and determine the impact of particulate matter (*PM*_10_) on each disease. To analyze the spatiotemporal correlations between allergic diseases (asthma, atopic dermatitis, and allergic rhinitis) and *PM*_10_ concentrations, we propose a multi-variable spatiotemporal graph convolutional network (MST-GCN)-based disease prediction model. Data on the number of patients were collected from the National Health Insurance Service from January 2013 to December 2017, and the *PM*_10_ data were collected from Airkorea during the same period. As a result, the proposed disease prediction model showed higher performance (*R*^2^ 0.87) than the other deep-learning baseline methods. The synergic effect of spatial and temporal analyses improved the prediction performance of the number of patients. The prediction accuracies for allergic rhinitis, asthma, and atopic dermatitis achieved *R*^2^ scores of 0.96, 0.92, and 0.86, respectively. In the ablation study of environmental factors, *PM*_10_ improved the prediction accuracy by 10.13%, based on the *R*^2^ score.

## Introduction

Allergic diseases (e.g., asthma, allergic rhinitis, atopic dermatitis) have continuously increased in prevalence and severity over the past 20 years [1]. Most patients suffering from these diseases have chronic symptoms and experience varying degrees of severity according to environmental changes (e.g., meteorological conditions and air pollution), which lead to a decrease in individual quality of life and an increase in social medical expenses. Therefore, various studies [2, 3] have been conducted to analyze the factors affecting allergic diseases to adjust for strategic health management.

The increased concentration of particulate matter (*PM*) generated by rapid urbanization and industrial advancement has emerged as a significant global concern within the realm of air pollution [4]. *PM*_10_ indicates solid or gaseous particulate matter suspended in the atmosphere with particle radii less than 10*μm*. Generally, the concentration of naturally occurring *PM*_10_ is only a small fraction of the overall concentration of *PM*_10_. As *PM*_10_ floats in the atmosphere, it endangers public health in the long term by causing lung cancer and cardiovascular and respiratory diseases [5, 6]. Specifically, because oral and respiratory routes refer to one of the major boundaries of the body, environmental changes greatly affect the homeostasis of the respiratory mucosae, which leads to allergic rhinitis and asthma [7]. Skin is also a common pathway for environmental contaminants to enter the body, making it vulnerable to atopic dermatitis [8]. Several epidemiological studies [9–15] have shown that exposure to ambient air pollutants is a major cause of increased hospitalization for allergic diseases. In addition, previous studies [16–18] found that an increase of 10*mg*/*m*^3^ in *PM*_10_ increased the risk of hospitalization for allergic rhinitis, atopic dermatitis, and asthma.

Although various studies have analyzed the relationship between *PM*_10_ concentrations and allergic diseases, their results have not been consistent. This is because the composition and concentration of *PM*_10_ are extremely variable and depend on various factors such as climate variability, emission sources, and geographical position [19, 20]. Previous epidemiological studies have attempted to identify high-risk patients based on traditional statistical techniques [21] (e.g., ARIMA and SARIMA), conventional machine learning models [22, 23], and time-series models [24] (e.g., recurrent neural networks (RNN) and long short-term memory (LSTM)). However, as the dynamic environmental factors in each region significantly influence the occurrence of these diseases, conventional time-series models cannot accurately predict the number of patients with allergic diseases.

While there has been a lot of recent research into using deep learning in medicine, most disease prediction research is based on analyzing patient-level chest X-rays, CT scans, and ultrasound audio and video to identify patients and diagnose diseases. Few studies have analyzed the epidemiology of disease at a demographic level. Among these, a previous study [21] used machine learning techniques to predict the number of patients who visited the emergency department of a particular hospital in order to identify demand for hospital services in advance. In addition, Wu et al. [25] used CNNs and LSTMs to extract spatiotemporal patterns to detect infectious disease outbreaks. The CNN based spatial pattern extraction methods have difficulty in expanding the receptive fields to predict over a large range of area. Recently, graph neural networks (GNNs) have been used to capture the spread of the COVID-19 pandemic, and spatiotemporal GNNs [26–28] in particular have shown reasonable sptiltemporal prediction performance from the perspective of disease spread. However, to the best of our knowledge, there are no existing studies that use GNN based prediction methods to predict the number of patients with allergic diseases that are not contagious and are highly influenced by dynamic ambient environmental factors. Therefore, this study aimed to predict the daily number of patients significantly influenced by *PM*_10_ concentrations.

**Fig 1:**
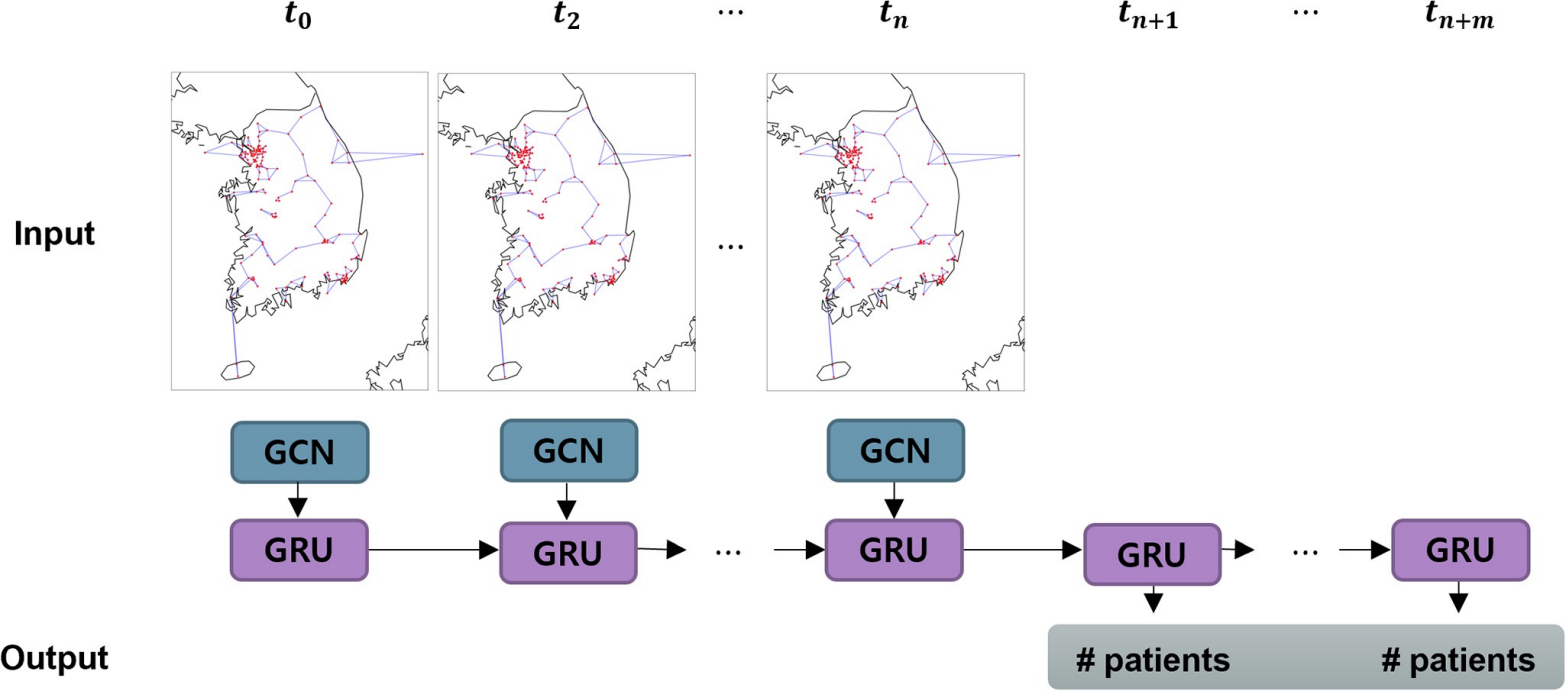
An architecture of the proposed disease prediction model. *n* and *m* indicate the length of observation sequences and prediction sequences of the temporal model, respectively.

Existing disease prediction methods have limitations in reflecting environmental factors with large variability between neighboring regions, owing to the aforementioned fluctuating characteristics of allergic disease distribution and *PM*_10_ concentration. To solve this problem, we propose a multivariable spatiotemporal graph convolutional network (MST-GCN) [29]-based prediction model to reflect the spatial distribution of *PM*_10_ around the target region based on the administrative district (si-gun-gu) in South Korea. Fig 1 shows the structure of the proposed disease prediction model illustrated in Fig 1. This model consists of a GCN layer [30] that extracts the regional diffusion process of *PM*_10_ concentration and daily number of patients, and a gated recurrent unit (GRU) layer [31] that captures the dynamic pattern of regional characteristics extracted from GCN. The performance of the proposed model was verified by showing meaningful results that could predict the daily number of patients with better accuracy (*R*^2^ 0.87) than other baseline deep learning models. In addition, our proposed model predicts the daily number of patients for up to 14 days in the future with stable accuracy. Therefore, in this study, we expect that the disease prediction model could be used to adjust strategic health management with continuous supplementation.

## Materials and methods

### Public health dataset

The disease data used in this study were sourced from the allergic disease database of the National Health Insurance Service https://nhiss.nhis.or.kr/bd/ab/bdabf001cv.do. These data provide the number of outpatients, inpatients, and emergency medical visits by residential area for daily patients diagnosed with asthma, atopic dermatitis, and allergic rhinitis. The patients were classified according to age and sex. The residential areas were in units of the administrative district (si-gun-gu), and average geocoding coordinates were provided for each unit. We used a database consisting of 5 years of data from 2013 to 2017, and this data was collected from 99% of Koreans enrolled in health insurance. This study aimed to predict the total number of daily patients in each area. Therefore, to extract the daily number of patients from the database, we preprocess the data to combine the number of patients divided by types of visits, age, and sex.

### Air quality dataset

The air quality data collected were hourly *PM*_10_ concentrations measured at 331 stations provided by Air Korea, managed by the Ministry of Environment https://www.airkorea.or.kr/web/last_amb_hour_data?pMENU_NO=123. The data were preprocessed to collect average daily *PM*_10_ concentrations. In addition, the concentration of *PM*_10_ was aggregated by averaging the concentrations at all observation stations belonging to a specific si-gun-gu. If the data collected at each station had a missing rate of more than 50 Finally, daily *PM*_10_ and public health data were collected for 161 si-gun-gu. We also used calendar variables such as year, month, and day as important features to reflect the seasonal and annual patterns of *PM*_10_ and disease in our spatiotemporal GNN methods.

### Spatiotemporal graph neural networks

As allergic diseases are influenced by environmental factors such as *PM*_10_, capturing spatial dependence is the main problem in predicting the number of patients with allergic diseases. A convolutional neural network (CNN) [32–34] can capture local spatial features when analyzing medical images or records represented by regular Euclidean data. However, the population of patients with allergic diseases in each region exhibits irregularly structured data, similar to gene interaction networks and chemical molecular structures, which means that they are graph-structured data rather than regular grid data to which CNNs can be applied. Recently, graph convolutional networks (GCN) [35–37] have been used to capture the structural features of graphs for various tasks, including medical diagnoses. A few studies [38] have conducted disease prediction using the GCN model, which considers comorbidity relationships among diseases, to discover new disease correlations among their study cohorts. Other studies [39] identified latent patients with a higher probability of developing diseases by considering the relationships between patients based on their electronic medical records (EMRs). In addition, Lu and Uddin [40] predicted chronic diseases based on GCN by reflecting the relationship between the patient and disease.

In this study, we focused on the population of patients between local regions. However, because the above studies focused on the probability of disease occurrence in individual patients, nodes in the graph defined as a specific disease or patient as an object have difficulty representing the characteristics of the number of patients across regions. Even in studies that conduct disease prediction tasks, the association between nodes cannot be defined consistently across all studies. Therefore, we defined our disease network and applied the spatiotemporal GCN model to predict the number of patients in each si-gun-gu at a particular period based on historical disease information and *PM*_10_ concentrations. Disease information indicated the number of patients with asthma, atopic dermatitis, and allergic rhinitis in each region. Therefore, the topological structure of disease propagation is described as *G* = (*V*, *E*), called the disease network, where *V* = {*v*_1_, *v*_2_, ⋯, *v*_*N*_} is the set of regions and *E* is the set of edges that reflect the k-nearest regions. The number of patients was regarded as an attribute of each node in the disease network. To cover the three allergic diseases, we defined attributes as a set of disease populations for each disease. The external variables affecting diseases, including *PM*_10_ concentration, were regarded as the remaining node attributes.

In summary, we can define the disease prediction task by learning the function *f*(⋅) based on the disease network *G* = (*V*, *E*), consisting of the static adjacency matrix *A* which considers the interregional correlation between the disease and *PM*_10_ and the dynamic node attributes *X* which reflect the temporal dependency of the number of patients. The prediction task is formulated as follows:

$$\begin{array}{c} <X_{\left(t+1\right)},\cdots ,X_{\left(t+l_{p}\right)}>=f\left(A,<X_{\left(t-l_{h+1}\right)},\cdots ,X_{t}>\right), \end{array}$$ (1)

where *X*_*t*_ denotes the node attribute at time *t*. Additionally, *l*_*h*_ and *l*_*p*_ indicate the historical and predicted sequence lengths, respectively. Therefore, the proposed model can perform long-term prediction tasks.

In a disease network, the GCN model can extract the topological relationship between the target si-gun-gu and its surrounding si-gun-gu by encoding the topological structure of the disease network and temporal node attributes. We stacked multiple GCN layers to update the node features to smooth the node features of adjacent nodes using a convolutional operator in the spectral space. The convolutional filters obtain spatial features between nodes by analyzing their first-order neighbors. The GCN layer is formulated as follows:

$$\begin{array}{c} H_{t}^{\left(n\right)}=\sigma \left(\hat{A}H_{t}^{\left(n-1\right)}\theta^{\left(n\right)}\right),H_{t}^{\left(0\right)}=X_{\left(t-l+1\right)}^{t},\hat{A}={\tilde{D}}^{-1/2}\hat{A}{\tilde{D}}^{-1/2}, \end{array}$$ (2)

where $A=\hat{A}+I$ indicates an adjacent matrix *A* and *I* ∈ *R*^*V*×*V*^ is an identity matrix. $\tilde{D}$ denotes the degree matrix of $\hat{A}$; $H_{t}^{\left(n\right)}$ denotes the feature matrix generated by the *n*^*th*^ layer at time *t*. $H_{t}^{\left(0\right)}$ denotes the initial node features at time *t*. *θ*^(*n*)^ represents the convolution filter in the *n*^*th*^ layer, and *σ*(⋅) indicates the activation function ReLU for nonlinear modeling. Thus, each GCN layer linearly transforms feature matrix $H_{t}^{\left(n-1\right)}$ according to $\hat{A}$. The number of patients is affected by the surrounding *PM*_10_ concentration, which may have a time-lag effect from the air pollutants to illness onset. T-GCN, which has difficulty handling multivariate data, is not sufficient to overcome long-term dependency. We apply the MST-GCN model to handle multiple node attributes and time points. The multiple-node attributes in the GCN layer at time *t* are formulated as <*X*_*t*−*l*+1_, ⋯, *X*_*t*_>.

To model the temporal dependencies between the disease and *PM*_10_ contexts, we fed a series of spatial features extracted from GCN into gated recurrent unit (GRU) layers. The proposed model can discover distinctive spatiotemporal correlations from temporal changes in the sequence of feature matrices $H_{1}^{\left(n\right)},\cdots ,H_{T}^{\left(n\right)}$. The GRU model is a variant of a recurrent neural network (RNN), which is widely used for processing time-series data. It consists of reset and update gates and has the advantages of a simple structure, few parameters, and fast learning speed. The reset gate controls the amount of information from previous time points, and the update gate controls the amount of past information that should be reserved to overcome the long-term dependency problem.

Disease prediction aims to make the prediction results approximate the actual number of patients as closely as possible. Therefore, the objective of the prediction model is to minimize the prediction error. The error was measured using the *L*_2_ loss, and the objective function can be formulated as follows:

$$\begin{array}{c} Loss=\|Y_{t}-\hat{Y_{t}}{\|}_{2}^{2}+{\lambda \|\theta \|}_{2}^{2}, \end{array}$$ (3)

where *Y*_*t*_ and $\hat{Y_{t}}$ are the real and predicted numbers of patients at time *t*, respectively, and λ is a hyperparameter controlling the regularization rate.

### Baseline methods

Although the Autoregressive Integrated Moving Average (ARIMA) model [21] is commonly used for time-series forecasting, we implemented the Seasonal ARIMA (SARIMA) model to exploit the underlying seasonality of the changing number of patients with allergic diseases. The model consisted of three parts: autoregression, difference, and moving average. This can be represented as *SARIMA*(*p*, *d*, *q*)(*P*, *D*, *Q*)_*s*_, where (*P*, *D*, *Q*) indicates the seasonal part and *s* represents the length of the seasonality. In addition, *p*, *d*, *q* and *P*, *D*, *Q* refer to the autoregression, difference, and moving average, respectively. In our experiments, we used *SARIMA*(1, 0, 1)(0, 1, 1)_14_ as the univariate SARIMA model.

Furthermore, we verified the superiority of our disease prediction model in terms of accuracy compared with deep-learning-based baseline methods, including GCN [30] (spatial), GRU [31] (temporal), and T-GCN [41] (spatiotemporal). As mentioned in the previous subsection, GCN can extract spatial features from the disease network by aggregating the node features with their neighborhoods. We implemented a two-layer GCN model to predict the future number of patients by analyzing $<\;X_{t-l_{h}+1},\cdots ,X_{t}\;>$ without considering the temporal order. GRU is one of the recurrent neural network (RNN) models that improves the long-term dependency problem of conventional RNN. We adopted a two-layer GRU model with 128 hidden units to perform our prediction task for each si-gun-gu by analyzing only temporal changes. The T-GCN combines GCN and GRU models designed to analyze networks with both static and dynamic features. We stacked two GCN layers to extract spatial features at each time point and added one GRU layer to predict the number of future patients by analyzing temporal changes. The MST-GCN-based disease prediction model proposed in the previous section is an improved model that reflects the time series of multiple variables (e.g., air pollution and disease population).

### Evaluation metrics

To evaluate disease prediction accuracy, we used five evaluation metrics: root mean square error (RMSE), mean absolute error (MAE), accuracy (ACC), coefficient of determination (*R*^2^), and explained variance score (var). When *Y*_*t*_ denotes the real number of patients and *Y*_*t*_ indicates the predicted number of patients at time *t*, the metrics can be formulated as follows:

$$\begin{array}{c} RMSE={\left[\frac{1}{T}\sum_{\forall t}{\left(Y_{t}-\hat{Y_{t}}\right)}^{2}\right]}^{\frac{1}{2}},MAE=\frac{1}{T}\sum_{\forall t}\left\|Y_{t}-\hat{Y_{t}}\right\|,\\ ACC=1-\frac{RMSE}{{\left\|Y\right\|}_{F}},R^{2}=1-\frac{\sum_{\forall t}{\left(Y_{t}-\hat{Y_{t}}\right)}^{2}}{\sum_{\forall t}{\left(Y_{t}-\bar{Y}\right)}^{2}},var=1-\frac{\sigma (Y-\hat{Y})}{\sigma (Y)}. \end{array}$$ (4)

where *T* indicates the total number of time points and *Y* is the average *Y*_*t*_. In addition, ‖ ⋅ ‖_*F*_ denotes the Frobenius norm and *σ*(⋅) indicates the variance. RMSE and MAE represent the average errors of the prediction models. ACC is the normalized error. In addition, *R*^2^ and *var* are correlation coefficients that measure the ability to predict the true data. Therefore, a smaller RMSE and MAE indicate better performance, and higher ACC, *R*^2^, and var indicate better performance.

## Results

In this section, we evaluate the predictive performance of the proposed model and validate the association between allergic diseases and *PM*_10_ concentrations. First, we verify the ability of our spatiotemporal MST-GCN model to predict the number of patients at the next time points in Section. We evaluated the results through a comparative analysis with baseline models, including a conventional regression model (e.g., SARIMA) and deep learning models (e.g., GCN, GRU, and T-GCN). Secondly, to clarify the influence of *PM*_10_ concentration on the prediction results, we compared the prediction accuracy by excluding *PM*_10_ concentration from the input variables in Section. Finally, we extend our investigation to evaluate the performance of the proposed model in long-term predictions, including prediction periods of 1, 7, and 14 days for each disease in Section.

### Effectiveness of the spatiotemporal analysis for disease prediction

This section evaluates our MST-GCN-based disease prediction model by comparing its accuracy with that of the baseline models. All the models were trained to predict the number of patients at time *t* by analyzing the previous *PM*_10_ concentrations and disease propagation patterns from *t* − 1 to *t* − 14. Table 1 presents the results for predicting the number of patients with all three diseases (i.e., all) and the results for predicting the number of patients with individual diseases (i.e., Asthma, Atopic dermatitis, and Allergic rhinitis).

**Table 1:** A performance comparison of the MST-GCN model with other baseline models. The bold font indicates models with the best performance on each metric.

Diseases	Metrics	SARIMA	GCN	GRU	T-GCN	MST-GCN
All	RMSE	-	0.08	0.04	0.03	**0.03**
	MAE	-	0.06	0.03	0.02	**0.02**
	ACC	-	0.39	0.70	0.74	**0.77**
	*R* ^2^	-	0.10	0.78	0.83	**0.87**
	*var*	-	0.10	0.79	0.84	**0.87**
Asthma	RMSE	0.04	0.08	0.03	0.03	**0.03**
	MAE	0.04	0.08	0.02	0.02	**0.02**
	ACC	0.78	0.42	0.81	0.83	**0.84**
	*R* ^2^	0.61	0.00	0.89	0.91	**0.92**
	*var*	0.62	0.00	0.90	0.91	**0.92**
Atopic dermatitis	RMSE	0.06	0.07	**0.03**	0.04	0.04
	MAE	0.05	0.05	**0.02**	0.03	0.03
	ACC	0.68	0.35	**0.75**	0.58	0.62
	*R* ^2^	0.53	0.00	**0.85**	0.59	0.65
	*var*	0.54	0.00	**0.86**	0.60	0.65
Allergic rhinitis	RMSE	0.03	0.07	0.03	0.02	**0.01**
	MAE	0.02	0.07	0.02	0.02	**0.01**
	ACC	0.68	0.35	0.71	0.79	**0.88**
	*R* ^2^	0.65	0.00	0.81	0.89	**0.96**
	*var*	0.66	0.00	0.85	0.90	**0.96**

For the multi-disease prediction results (i.e., all diseases), the MST-GCN model outperformed the other baseline methods for every evaluation metric. By contrast, the GCN model exhibited the lowest accuracy of 0.39. Compared with the GRU model, which showed an accuracy of 0.70, the number of future patients depended more strongly on the historical data trained by GRU rather than on the spatial distribution of the patient population learned by GCN. Convolutional networks alone are insufficient for extracting the spatial patterns revealed in air pollution and disease data. However, T-GCN, which is a combination of graph convolutional and recurrent layers, has an accuracy of 0.74, which is higher than GRU. Therefore, we verified that spatiotemporal analysis predicted the number of patients with allergic diseases more effectively than individual analyses. Consequently, the best performance of the MST-GCN model suggests that the synergistic effect of spatial and temporal analysis, reflecting the correlation between allergic diseases and *PM*_10_ concentrations, improves the prediction accuracy. As SARIMA cannot handle multivariate prediction tasks, we could not evaluate the accuracy of the conventional method in this case. Consequently, these values have been left blank in Table 1.

For individual disease, the accuracy of the GRU, T-GCN, and MST-GCN models are 6.18(±2.91)%, 2.63(±12.89)%, and 7.69(±15.66)% higher than that of the SARIMA model. Because the SARIMA model performs modeling by calculating the error of each node and then averaging it, if there are fluctuations in the time-series data, the total error also increases. This makes it difficult for the SARIMA model to handle abnormal data. Nevertheless, the SARIMA model performed better than the GCN model for all individual disease cases. The lower performance of the GCN model was because the graph convolutional layers only reflected the spatial features and ignored the influence of historical disease data on the number of patients.

Both results, predicting the number of patients with asthma and allergic rhinitis, are similar to the multi-disease prediction results (i.e., all diseases) described above in terms of performance differences between the models. In contrast, in the case of atopic dermatitis, the GRU model exhibited the best performance. The MST-GCN-based model, which combines spatial and temporal features with *PM*_10_ concentrations, underperformed the GRU model, which focuses solely on temporal features. Spatial patterns and multivariate analyses negatively impacted the prediction of the number of patients with atopic dermatitis. For asthma and allergic rhinitis, the number of patients in one si-gun-gu group was closely correlated with the number of patients in neighboring regions that shared similar environmental characteristics, indicating a strong spatial correlation. However, for atopic dermatitis, the spatial correlation is weaker and more difficult to measure because an increase in the number of patients in one si-gun-gu may or may not indicate changes in other regions. Therefore, in the case of atopic dermatitis, graph convolutional-based models cannot extract distinct spatial features according to the number of patients in adjacent si-gun-gu. This difficulty remains even when multivariate analysis, including *PM*_10_ concentration, is considered.

Comparing the best performance by disease, the prediction accuracies for asthma (0.84) and allergic rhinitis (0.88) were higher than those for predicting multiple allergic diseases simultaneously (0.77). This result is due to the fact that predicting multiple diseases is a more complex problem than predicting a single disease. Nevertheless, the lower performance in predicting atopic dermatitis than in predicting multiple diseases simultaneously suggests that there are limitations in learning the number of patients using spatiotemporal analysis. We can assume that for atopic dermatitis, the detailed characteristics of a region are more important than the interactions between neighboring regions. Allergic rhinitis has the highest prediction accuracy (0.88). This result indicates that the spatiotemporal analysis and the relationships between diseases and *PM*_10_ concentrations have the most significant impact on the number of patients with allergic rhinitis.

### Effectiveness of the multi-variable spatiotemporal graph neural network

**Fig 2:**
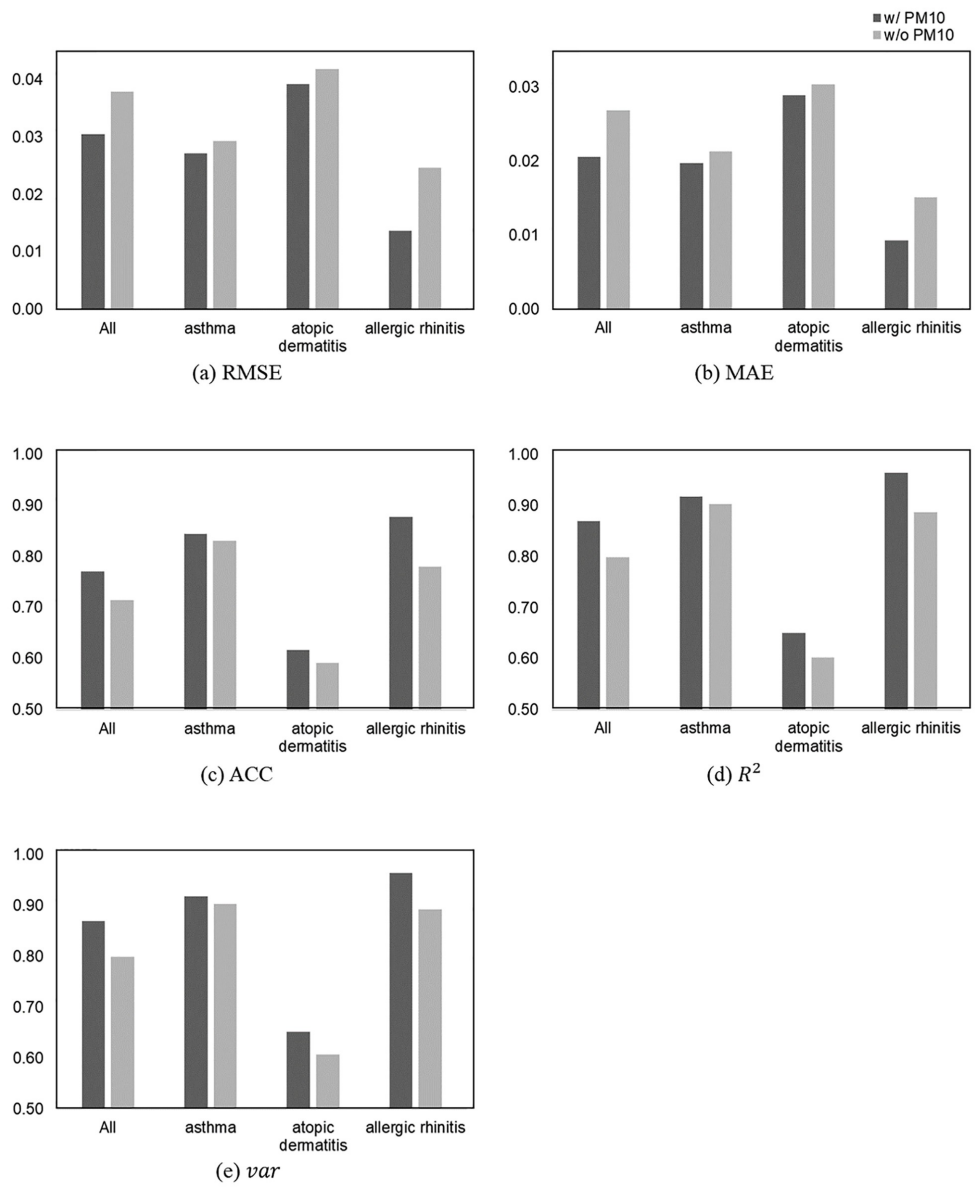
A comparison of the prediction performance in MST-GCN with and without *PM*_10_ concentrations in the input data. X-axes of the plots refer to each disease, and Y-axes correspond to the evaluation metrics. **(a)**-**(e)** indicate the five evaluation metrics.

To verify the effectiveness of multi-variable input data in our disease prediction model, we conducted an ablation study, the results of which are shown in Fig 2. All experiments were conducted on the MST-GCN-based model, and the results were obtained from the output of the prediction one day in advance.

To evaluate the contribution of the *PM*_10_ concentrations to the disease prediction model, we designed two variants: MST-GCN with *PM*_10_ concentrations and MST-GCN without *PM*_10_ concentrations in the input data. In all experimental cases and evaluation metrics, predictions with *PM*_10_ concentrations outperformed predictions without *PM*_10_ concentrations. In Fig 2(d), where the difference in performance is the greatest, the *R*^2^ values for predicting all diseases, asthma, atopic dermatitis, and allergic rhinitis with *PM*_10_ concentrations increased by 5.70%, 1.27%, 2.90%, and 6.94%, respectively. Therefore, we can assume that all allergic diseases are affected by spatiotemporal characteristics of *PM*_10_ concentrations, among which allergic rhinitis is the most affected.

Asthma has a higher accuracy than the multi-disease prediction results (i.e., all diseases), whereas the percentage improvement in accuracy is lower than the multi-disease results. In the case of asthma, the number of patients in neighboring regions and the temporal pattern may have a more significant influence than *PM*_10_ concentrations. Atopic dermatitis had the lowest accuracy, both with and without *PM*_10_ concentrations. Nevertheless, including air pollution data significantly improved the model’s accuracy. With these results, we can validate the positive role of *PM*_10_ concentrations in our disease prediction model, despite the limitations of spatiotemporal analysis for atopic dermatitis.

### Per-2weeks evaluation results

Regarding the timing of exposure and disease development, exposure to *PM*_10_ is associated with new-onset allergic diseases and has a persistent effect, with a time lag of a few months [16]. *PM*_10_ can remain in the human body for weeks after exposure without triggering an immune response in individuals with allergic diseases. Therefore, we compared the accuracy of the model for different prediction periods (Table 2). This experiment aimed to determine the temporal relationship between *PM*_10_ concentration and allergic diseases. In addition, we examined the practicality of our spatiotemporal analysis for long-term predictions. The range of long-term predictions for the GNN-based approach was typically within 12 future time steps. Distinct inputs must traverse complex spatiotemporal graph neural network structures that are far from effective for extracting temporal correlations. In our experiment, we extend the prediction period to 14 days. We conducted an experiment using the MST-GCN model, which exhibited the best performance by capturing the spatiotemporal characteristics described in the previous section.

**Table 2:** A performance comparison of the MST-GCN model with other baseline models. The bold font indicates models with the best performance on each metric.

	Diseases											
	All			Asthma			Atopic dermatitis			Allergic rhinitis		
Prediction length	1day	7days	14days	1day	7days	14days	1day	7days	14days	1day	7days	14days
RMSE	0.03	0.03	**0.03**	0.03	0.03	**0.02**	0.04	**0.03**	0.04	**0.01**	0.02	0.02
MAE	0.02	0.02	**0.02**	0.02	0.02	**0.02**	0.03	**0.02**	0.03	**0.01**	0.01	0.01
ACC	0.77	0.77	**0.78**	0.84	0.84	**0.86**	0.62	**0.68**	0.64	**0.88**	0.85	0.85
*R* ^2^	0.87	0.87	**0.87**	0.92	0.92	**0.93**	0.65	**0.75**	0.69	**0.96**	0.95	0.94
*var*	0.87	0.87	**0.88**	0.92	0.92	**0.93**	0.65	**0.75**	0.69	**0.96**	0.95	0.94

When predicting multiple diseases (i.e., all diseases), the accuracy of the 14-day prediction was slightly higher than that of the 1-day or 7-day predictions. However, we determined that the model showed a constant performance from 1-day to 14-day predictions, as the difference in accuracy according to the prediction period was less than 0.01%. In general, long-term predictions are sensitive to errors, making it difficult to predict the number of patients with complex and dynamic spatiotemporal correlations. The error can propagate through the correlations to reach the spatiotemporal positions in every future prediction, resulting in a butterfly effect of significant error propagation after each time step. Despite these challenges, the accuracy of the proposed model remained consistent for long-term predictions. This result validated the strong temporal correlation between allergic diseases and exposure to *PM*_10_, which enables accurate long-term disease prediction. Moreover, the MST-GCN model can address long-term dependency problems by learning temporal changes in spatial features using multiple variables, including *PM*_10_ concentrations. The experimental results indicated that the proposed model can effectively understand and extract the correlation between air pollution and allergic diseases.

Asthma, atopic dermatitis, and allergic rhinitis showed the highest accuracies for the 14-day, 7-day, and 1-day predictions, respectively. The accuracy of asthma prediction, similar to that of multiple disease prediction (i.e., all diseases), was higher at extended prediction periods. The accuracy of predicting the number of patients with asthma (0.86) is higher than that of predicting multiple diseases (0.78) in a 14-day forecast. After exposure to *PM*_10_, asthma symptoms may occur after a time lag rather than immediately. The high accuracy indicates that the temporal pattern of hospital visits for asthma following exposure to *PM*_10_ was consistent. Therefore, we can assume that the time lag between exposure and asthma onset has a constant pattern of less than two weeks.

In contrast, in the case of allergic rhinitis, the 1-day prediction showed the best performance. One reason for this is that including irrelevant temporal information in historical data introduces noise into the prediction model, leading to increased error propagation in the prediction of allergic rhinitis. Consequently, it performs worse for long-term predictions. The RMSE increased by 0.01, and the accuracy decreased by 0.03 for the 1-day and 14-day predictions. However, these changes are considered minimal. Therefore, it can be concluded that the symptoms of allergic rhinitis are delayed after exposure to *PM*_10_, but the response is relatively immediate compared with other allergic diseases. However, it cannot be assumed that allergic rhinitis has a weak temporal correlation because allergic rhinitis shows the best accuracy in every period compared with the other cases.

The significantly lower accuracy of predicting the number of atopic dermatitis patients compared to other diseases indicates that this disease has a weak spatial correlation with the environment in neighboring si-gun-gu, as mentioned in the previous subsection. To validate the temporal correlation, we compared the accuracy with the length of the prediction period and found that atopic dermatitis had the highest accuracy for a 7-day forecast. Therefore, we verified that the temporal pattern of the surrounding environment strongly influences atopic dermatitis. The 7-day forecast had better prediction performance than the 14-day forecast. The temporal correlation between disease and air pollution was more significant during the 7-day period than during the 14-day period. These results indicate that the prediction period with high performance differed for each disease. Although *PM*_10_ concentrations affect all allergic diseases, we assume that the time lag affected by *PM*_10_ concentrations may be different for each disease.

## Discussion

In this section, we analyze the experimental results from a medical perspective. First, we provide a medical interpretation of the experimental results in the previous section, focusing on the spatiotemporal impact of the environment on the three allergic diseases. We then analyze the characteristics of each disease. In addition, we also discuss the significance of our MST-GCN-based approach compared to statistical-based disease prediction, which is widely used in medical analysis, and clarify the limitations of our study.

### Characteristics of the allergic diseases

In Table 1, MST-GCN exhibited the best performance compared to the baseline models. Among the allergic diseases, the predictability was excellent in the following order: allergic rhinitis, asthma, and multiple diseases. In other words, allergic rhinitis and asthma have significant spatial and temporal correlations with the distribution of disease propagation and *PM*_10_ concentrations. However, atopic dermatitis has the highest predictive accuracy in time-series analysis (GRU), and its predictive performance deteriorates in models that include GCN layers for learning spatial patterns (GCN, T-GCN, and MST-GCN). The increase in the number of patients with atopic dermatitis is generally influenced by the season rather than the region. In addition, atopic dermatitis is influenced more by genetic and immunological factors than by *PM*_10_ concentrations [42]. Atopic dermatitis has various causes, including an impaired skin barrier and abnormal immune reactions [43]. These multiple etiologies of atopic dermatitis are responsible for the weak spatial correlation, where an increase in the number of patients in one si-gun-gu may or may not indicate changes in neighboring regions.

As shown in Table 2, we verified that the performance in predicting the number of patients was excellent for both short- and long-term predictions. Air pollutants affect allergic diseases by increasing oxidative stress and inflammatory responses [44]. The progression of this inflammatory response increases the levels of intracellular inflammatory mediators, amplifies hypersensitivity, and induces allergic diseases [45]. Allergic diseases are characterized by immunoglobulin E (IgE)-mediated hypersensitivity, and symptoms usually appear within a few hours of sensitization. Therefore, disease prediction showed high performance from 1 day. Asthma has a high prediction accuracy at 14 days, and it appears that it takes a relatively long time to induce airway hypersensitivity after bronchial epithelial cells are sensitized to *PM*_10_, and the symptoms are maintained for a relatively long time. Allergic rhinitis is expected to be easily predicted, even on the first day, because nasal mucosal cells, which are close to the external exposure and are easily sensitized to *PM*_10_, become rapidly sensitized. Atopic dermatitis is less predictable than asthma or rhinitis. It seems that atopic dermatitis is less sensitive to *PM*_10_ than asthma or rhinitis. The accuracy of the 7-day prediction was high because the skin cell hypersensitivity reaction seemed to occur more slowly than in the nasal mucosa and bronchial epithelial cells. The skin cell barrier reacts slower to allergens than to the nasal or respiratory mucosa. As a result, the proposed model can understand each disease characteristic based on historical disease data with the surrounding *PM*_10_ concentrations. If the number of patients can be predicted up to a certain point in time, an appropriate medical supply can be provided and medical preparations can be established, as in the case of corona. This allowed us to warn susceptible individuals. Consequently, our proposed disease prediction model has a higher performance than existing models such as the susceptible, exposed, infection, removed (SERI) and autoregressive integrated moving average (ARIMA) models, because the deep learning model can extract nonlinear patterns and compensate for the limitations of statistical analysis.

### Significance of the deep-learning analysis in medicine

Most existing studies investigating the relationship between air pollution and allergic diseases have used observational, cross-sectional, and longitudinal cohort studies to avoid erroneous inferences based on grouped individuals. However, population approaches allow us to understand the complicated health effects of *PM*_10_ concentrations. Therefore, in this study, we proposed an MST-GCN-based disease prediction model to automatically analyze the spatiotemporal pattern of disease propagation and predict the number of patients with high accuracy. The impact of environmental factors and the extent of spatiotemporal dependencies on the development of allergic diseases are still under debate. Nevertheless, this study discovered the crucial role of *PM*_10_ concentration in predicting the number of patients in an AI manner. In addition, we verified that the spatiotemporal correlation between the disease and *PM*_10_ concentration differs depending on the disease by comparing the prediction accuracies of different models for each disease. These results will help to derive cost-effective environmental health policies to reduce the burden of allergic diseases.

### Limitations

In this section, we clarify the several limitations of this study. First, as this study focused on predicting the number of daily patients using si-gun-gu, we excluded patient-specific characteristics such as age and sex. The total number of patients per region was calculated as the sum of the number of patients according to age and sex. However, age [46] and gender [47] differences might also influence disease outcomes in response to air pollutant exposure. In future research, we will attempt to predict the number of patients by considering the groups of patients and analyzing the impact of air pollution on allergic diseases according to sex and age. In addition to *PM*_10_, other air pollutants [48, 49] such as ozone (*O*_3_), nitrogen dioxide (*NO*_2_), sulfur dioxide (*SO*_2_), and carbon monoxide (*CO*) also affect allergic diseases and can be influenced by a variety of risk factors, including socioeconomic and environmental variables. Although this study did not include other external factors, their combined effect could explain why a prediction accuracy of 100% could not be achieved. Therefore, we need to extend our method to include other risk factors. Additionally, the severity of the disease or information about individual patients was not collected in sufficient quantities to train the deep-learning models. This limitation makes it difficult to conduct specific analyses.

## Conclusions

We used the population numbers of representative allergic diseases, such as asthma, allergic rhinitis, and atopic dermatitis, and *PM*_10_ concentrations as input data for a disease prediction model. The number of patients with allergic diseases was predicted as model output. To deal with spatiotemporal correlations between allergic diseases and *PM*_10_ concentrations, we proposed a MST-GCN-based disease prediction model. The predictive ability is excellent in all models at 1, 7, and 14 days (*R*^2^ 0.65-0.96). In all allergic disease groups, the 14-day prediction was better than that of the other days (*R*^2^ 0.87). The number of patients with asthma had the highest accuracy in the 14-day long-term prediction (*R*^2^ 0.93). In the case of allergic rhinitis, the best performance was shown in 1-day prediction (*R*^2^ 0.96). The number of patients with atopic dermatitis showed the best performance in the 7-day prediction (*R*^2^ 0.75).
